# Endothelial Cell Proliferation in Swine Experimental Aneurysm after Coil Embolization

**DOI:** 10.1371/journal.pone.0089047

**Published:** 2014-02-14

**Authors:** Yumiko Mitome-Mishima, Munetaka Yamamoto, Kenji Yatomi, Senshu Nonaka, Nobukazu Miyamoto, Takao Urabe, Hajime Arai, Hidenori Oishi

**Affiliations:** 1 Department of Neurosurgery, Juntendo University School of Medicine, Tokyo, Japan; 2 Department of Neuroendovascular Therapy, Juntendo University School of Medicine, Tokyo, Japan; 3 Department of Neurosurgery, Juntendo University Urayasu Hospital, Chiba, Japan; 4 Department of Neurology, Juntendo University Urayasu Hospital, Chiba, Japan; University of South Florida, United States of America

## Abstract

After coil embolization, recanalization in cerebral aneurysms adversely influences long-term prognosis. Proliferation of endothelial cells on the coil surface may reduce the incidence of recanalization and further improve outcomes after coil embolization. We aimed to map the expression of proliferating tissue over the aneurysmal orifice and define the temporal profile of tissue growth in a swine experimental aneurysm model. We compared the outcomes after spontaneous thrombosis with those of coil embolization using histological and morphological techniques. In aneurysms that we not coiled, spontaneous thrombosis was observed, and weak, easily detachable proliferating tissue was evident in the aneurysmal neck. In contrast, in the coil embolization group, histological analysis showed endothelial-like cells lining the aneurysmal opening. Moreover, immunohistochemical and morphological analysis suggested that these cells were immature endothelial cells. Our results indicated the existence of endothelial cell proliferation 1 week after coil embolization and showed immature endothelial cells in septal tissue between the systemic circulation and the aneurysm. These findings suggest that endothelial cells are lead to and proliferate in the former aneurysmal orifice. This is the first examination to evaluate the temporal change of proliferating tissue in a swine experimental aneurysm model.

## Introduction

Endovascular detachable coil treatment of intracranial aneurysms is an effective and increasingly popular non-invasive alternative to surgical clipping. In patients with ruptured intracranial aneurysms, endovascular intervention with detachable platinum coils can improve the chances of independent survival compared with neurosurgical intervention to clip the neck of the aneurysm [1]–[3]. The benefits of endovascular therapy for unruptured cerebral aneurysms have not been demonstrated, as the natural history of unruptured cerebral aneurysms has not been clearly defined. Nonetheless, when compared with surgical clipping, endovascular therapy of unruptured aneurysms is associated with decreased risk of adverse outcomes and in-hospital death, lower hospital charges, and shorter hospital stays [4]. In future, the indications for and popularity of endovascular therapy is expected to increase further as better devices are developed.

One of the substantial shortcomings of endovascular therapy is the technical problem of aneurysm recanalization [5], [6]. Recanalization due to coil compaction and aneurysmal regrowth is typically a consequence of the volume embolization rate at the time of treatment, choice of device material, localization of an aneurysmal remnant and hydromechanic factors [7]. Recanalization should be avoided, as there is a high risk of aneurysmal rupture after endovascular therapy.

There are a few reports of pathological evaluations of aneurysms after endovascular coil treatment. They suggest that a stable aneurysmal thrombosis is achieved if an endothelium-lined layer of connective tissue forms between the aneurysm and parent artery after embolization [8], [9]. Swine models are used as a basis for cerebral aneurysm research in many institutions, because aneurysm sizes are similar to those of humans and can be easily evaluated by angiography. The hemodynamic, physiologic, and histologic changes in experimental aneurysms in swine more closely reflect those observed humans than in other animal models. Furthermore, the swine model is useful for preclinical testing of new devices before research use in humans and ultimately in clinical practice. In particular, the swine model permits the effect of drugs and devices on the speed of endothelialization to be evaluated. There are many histopathological reports of aneurysm models [10], however, the time course of spontaneous thrombosis after Guglielmi detachable coil (GDC) embolization in swine aneurysms has not been reported.

We examined the expression profile of proliferating tissue covering the aneurysmal orifice using histochemical and morphological techniques in a swine model of sidewall-type cerebral aneurysm after coil embolization and spontaneous thrombosis.

## Materials and Methods

### Sidewall Aneurysm Model

All animal procedures described in this report were approved by the Animal Care Committee of Juntendo University. Adult male and female Landrace-Yorkshire-Duroc swine weighing 40 to 50 kg were obtained from the National Livestock Breeding Center Ibaraki Station (Ibaraki, Japan) and maintained on a 12-hour light/dark cycle with free access to food and water. Thirty-six experimental aneurysms were surgically created in 18 swine divided two groups – the intervention group underwent coil embolization and the control group did not. Sidewall aneurysms were created in both common carotid arteries as described previously [11], [12]. Briefly, the animals were anesthetized with 36.8 mg/kg ketamine hydrochloride (Daiichi Sankyo Co., Ltd., Tokyo, Japan) and 5.3 mg/kg xylazine (Bayer Healthcare, Leverkusen, Germany) administered intramuscularly and anesthesia was maintained with mechanical ventilation and inhalation of 1.5–2.0% isoflurane in 35% oxygen and 65% nitrogen after endotracheal intubation. The left external jugular vein was exposed and isolated by means of a midline neck incision using sterile technique. Two 6 mm lengths of venous pouch used for making the aneurysm dome were harvested and placed in sterile saline. Then, the left carotid artery was exposed, cleaned of adventitia and clamped with clips proximally and distally. After achieving stasis in this isolated segment, an approximately 2.8 mm diameter arteriotomy was made by aortic punch (Premium aortic punch; Teleflex Medical Japan, Tokyo, Japan) and an end-to-side anastomosis (vein to artery) was performed using 7-0 Prolene interrupted sutures (Ethicon Inc., Somerville, NJ). When we were satisfied that there was no major bleeding or leakage from anastomosis on inspection and using cineangiography as described Yatomi *et al*. [13], the proximal clamp was released and blood flow was re-established through the artery and aneurysm. A similar technique was used to create an aneurysm in the right common carotid artery. The stump of venous pouch was clamped with a clip to allow thrombectomy if the aneurysm had spontaneously thrombosed during the procedure. In the coil embolization group, the resulting aneurysms were immediately embolized with platinum coils.

### Embolization Procedure

Spontaneous thrombosis is common in the swine aneurysm model [14], [15], therefore, coil embolization was performed within 12 hours of the aneurysms being created. A cut-down was performed to gain percutaneous access to the right common femoral artery. A 4Fr sheath (Super sheath, Medikit, Miyazaki, Japan) was placed in the artery and connected to a continuous heparinized saline flush. A 4 Fr guiding catheter (JNS Type I, Medikit) was placed over an angled guidewire (Radifocus Guidewire, Terumo, Tokyo, Japan) and advanced into the proximal right common carotid artery. A microcatheter (Excelsior™ SL-10 Microcatheter, Boston Scientific/Target Therapeutics, Fremont, CA) was then inserted coaxially through the guiding catheter (Transend™ Guidewires, Boston Scientific/Target Therapeutics) into the aneurysmal cavity. Cineangiography was performed after injection of 5 ml iodinated contrast medium (Iopamiron Inj. 300, Bayer Healthcare). Aneurysms were packed as densely as possible with platinum coils (GDC™ Detachable Coil, Stryker, Tokyo, Japan).

### Harvesting Procedures

Swine were sacrificed 1, 2 or 4 weeks after the procedure (n = 3 for each group) after cineangiographic assessment. At sacrifice, the swine were deeply anesthetized with an intravenous injection of a lethal dose of pentobarbital (Kyoritsu Seiyaku Corporation, Tokyo, Japan). The aneurysm and parent artery were removed immediately *en bloc* and fixed for 48 hours in either 4% paraformaldehyde or 2.5% glutaraldehyde in phosphate-buffered saline (PBS) at 4°C. The gross appearance of the neck of the aneurysm was carefully examined and photographed. The metallic coil fragments were carefully removed from the aneurysm under a dissecting microscope.

### Histology

Following removal of all coil fragments, aneurysms were fixed in 4% paraformaldehyde for 2–5 days, embedded in paraffin, and sectioned at a thickness of 3 µm in a coronal orientation, allowing long-axis sectioning of the aneurysm neck. At least two sections from each block were stained with hematoxylin and eosin (H&E), AZAN, and a selection of the immunohistochemical stains described below for conventional histopathologic evaluation.

### Immunohistochemistry

After incubation in 3% H_2_O_2_ followed by blocking in 10% normal goat serum (Dako Corporation, Carpentaria, CA) in PBS, the sections were immunostained overnight at 4°C with antibodies against von Willebrand factor (vWF, dilution 1∶200; Dako), platelet endothelial cell adhesion molecule-1 (PECAM-1; dilution 1∶50, Santa Cruz Biotechnology, Santa Cruz, CA), proliferating cell nuclear antigen (PCNA; dilution 1∶200, Dako), CD34 antibody (dilution 1∶100; Abcam, Cambridge, MA). The sections were then treated with secondary antibodies (Vector Laboratories, Burlingame, CA). Immunoreactivity was visualized using the avidin-biotin complex method (Vectastatin ABC kit, Vector Laboratories) and developed with diaminobenzidine, followed by counterstaining with Gill hematoxylin (Sigma-Aldrich, St. Louis, MO).

### Transmission Electron Microscopy (TEM)

The aneurysms that had been immersed in 2.5% glutaraldehyde solution were processed for examination by TEM. The aneurysms were further cut into smaller pieces (1×2 mm^2^) and washed in PBS. The pieces were subsequently fixed in 2% osmium tetroxide for 2 hours at 4°C, dehydrated with graded concentrations of ethanol, and placed in resin for 4 days at 60°C. Semi-thin sections were stained with toluidine blue. Ultrathin sections were stained with uranyl acetate and lead citrate, placed on foamer-coated copper grids and examined under a JEM-1230 electron microscope (JEOL, Tokyo, Japan) at 80 kV.

### Cell Counts and Statistical Analysis

Values presented are expressed as mean ± standard error (SEM). Each group consisted of six aneurysms. Analysis of variance (ANOVA) and subsequent *post hoc* Fisher protected least significant difference tests were used compare differences between the groups. A *P*-value less than 0.05 represented a statistically significant difference.

## Results

### Angiographic Findings

**Figure 1:**
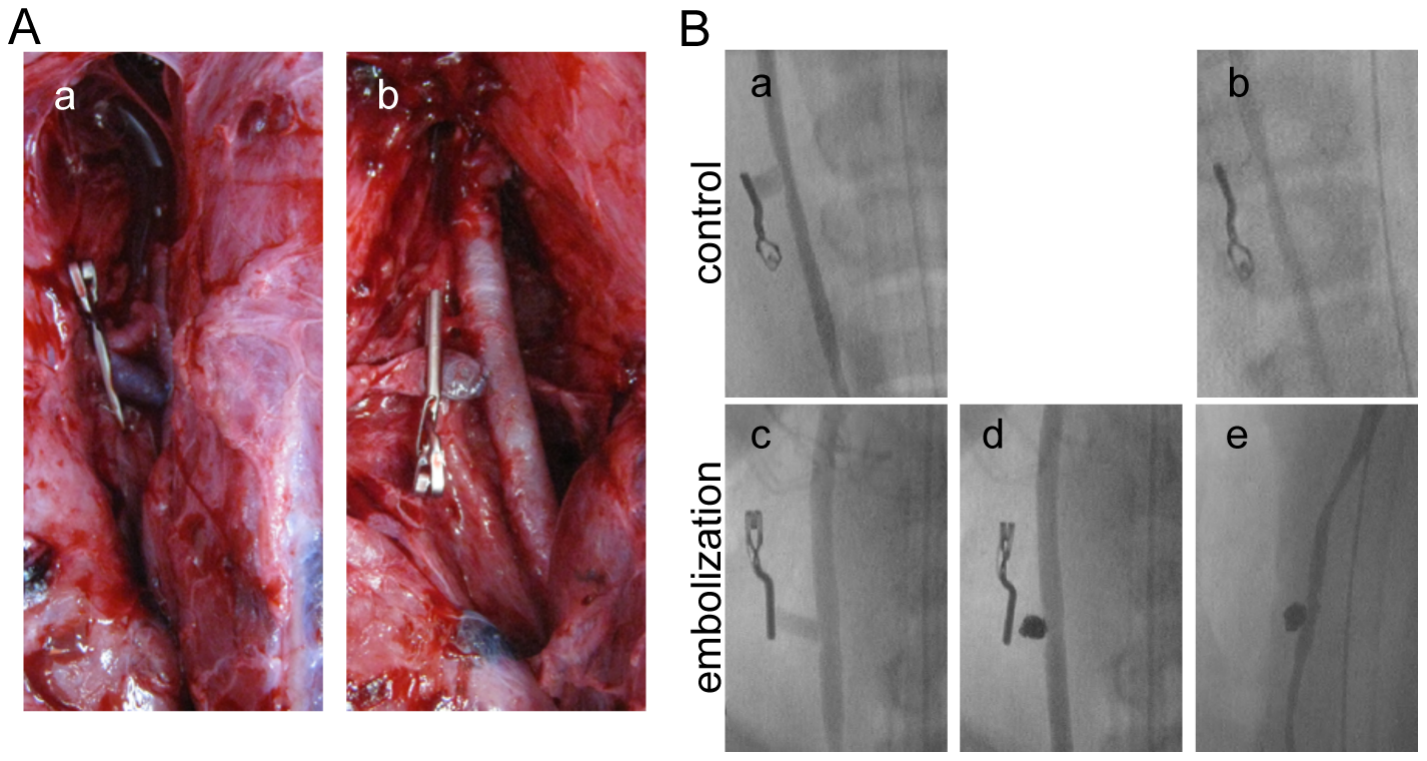
Intra- and postoperative angiograms. (**A**) Intraoperative views of the sidewall-type aneurysm a) before and b) after coil embolization. (**B**) a and c) intraoperative angiograms; d) angiogram obtained immediately after coil embolization; and b and e) angiogram obtained 1 week after surgery.

None of the swine died during the experiment, and all were confirmed to have aneurysms in both common carotid arteries (Figure 1A). All aneurysms were embolized without any technical complications and packed as tightly as possible with platinum coils. Cineangiography was undertaken before and after coil embolization, and before sacrifice in the embolization and control groups. Representative cineangiograms are shown in Figure 1B. None of the aneurysms showed angiographic evidence of coil compaction or recanalization.

### Macroscopic Findings

**Figure 2:**
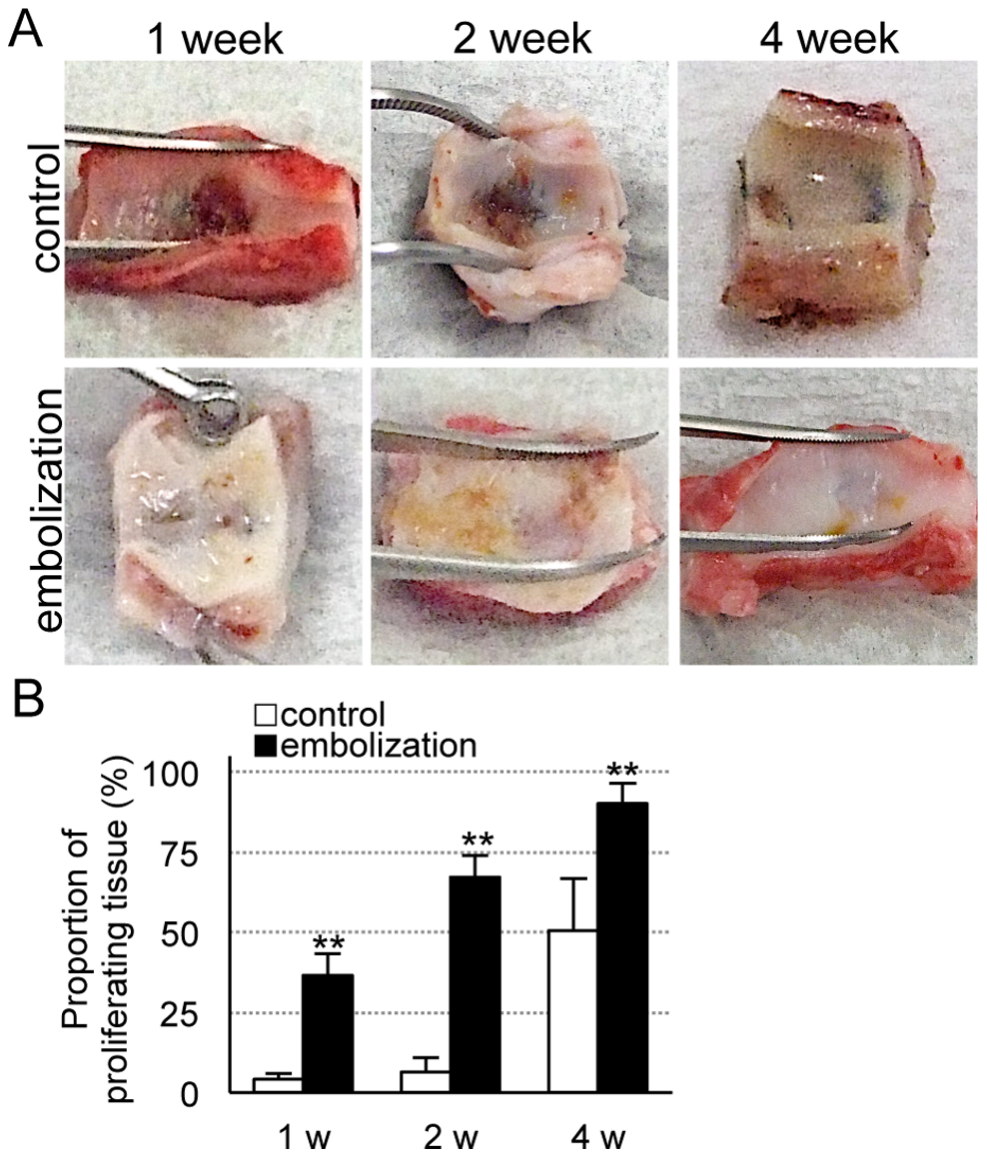
Evaluation of the aneurysmal orifice. (**A**) Photomicrographs of aneurysmal orifices 1, 2 and 4 weeks after the operation. (**B**) The proportion of proliferating tissue covering the aneurysmal orifice in the embolization and control groups. Data are mean ± SEM of three swine in each group. *P<0.05, **P<0.001, compared with the control group.

Macroscopic findings showed thin to moderately thick fibrous neointimal membranes covering the aneurysmal orifice harvested at 1 and 2 weeks, and very thick fibrous neointimal membranes covering the aneurysms harvested at 4 weeks after coil embolization, compared with aneurysms that were not coiled (Figure 2A). The area of the covering membrane was significantly greater in the coil embolization group (*P*<0.001, Figure 2B).

### Histological Findings

**Figure 3:**
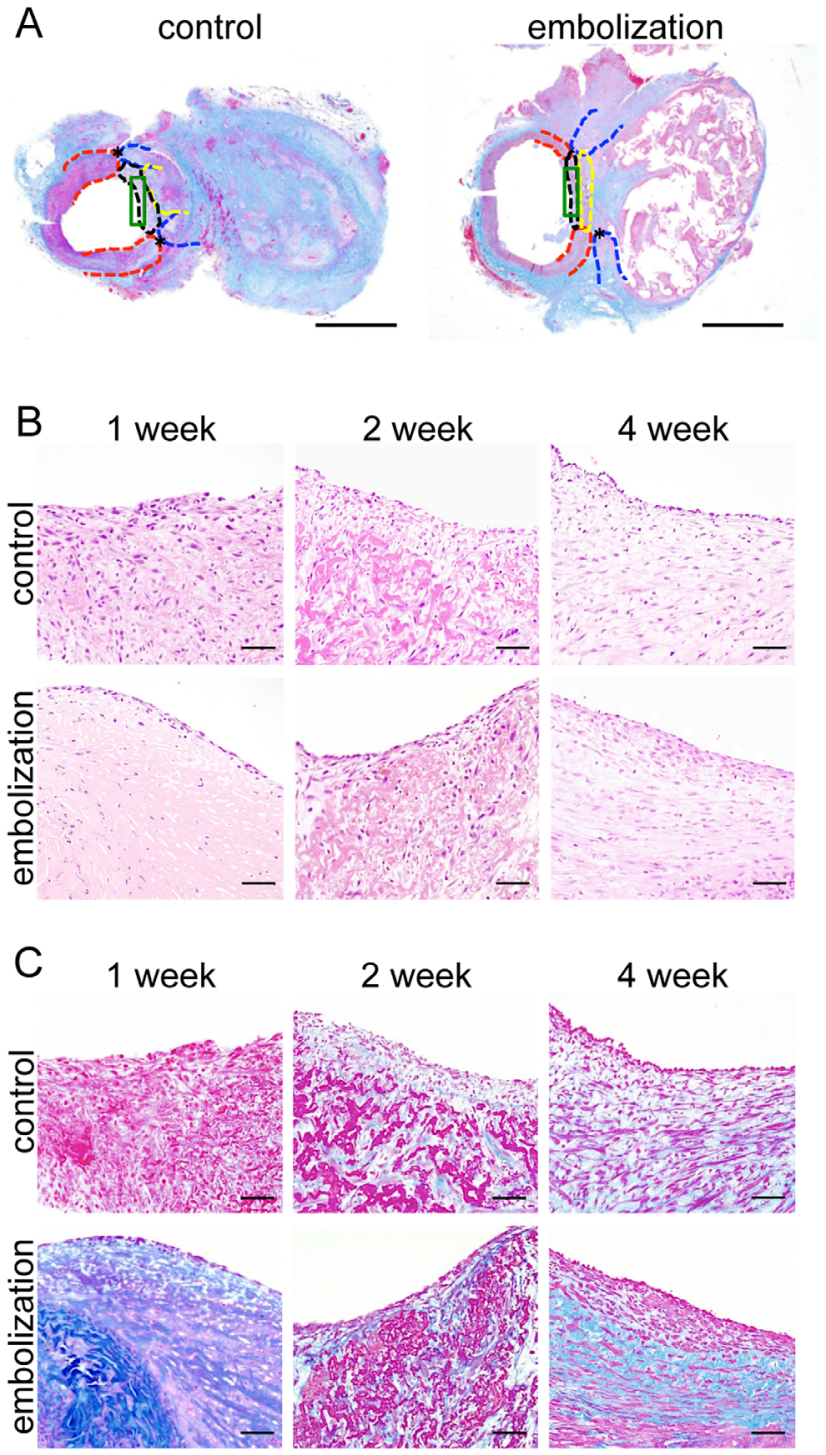
Histology of proliferating tissue. (**A**) Photomicrographs of AZAN stained tissue 4 weeks after surgery: red dotted line, artery; blue dotted line, vein; yellow dotted line, thrombosis; black dotted line, proliferating tissue; green enclosure, evaluation area. Scale bars = 2 mm. (**B**) Photomicrographs of H&E stained tissue 1, 2 and 4 weeks after surgery in the embolization and control groups. Scale bars = 50 µm. (**C**) Photomicrographs of AZAN stained tissue 1, 2 and 4 weeks after surgery in the embolization and control groups. Scale bars = 50 µm.

**Figure 4:**
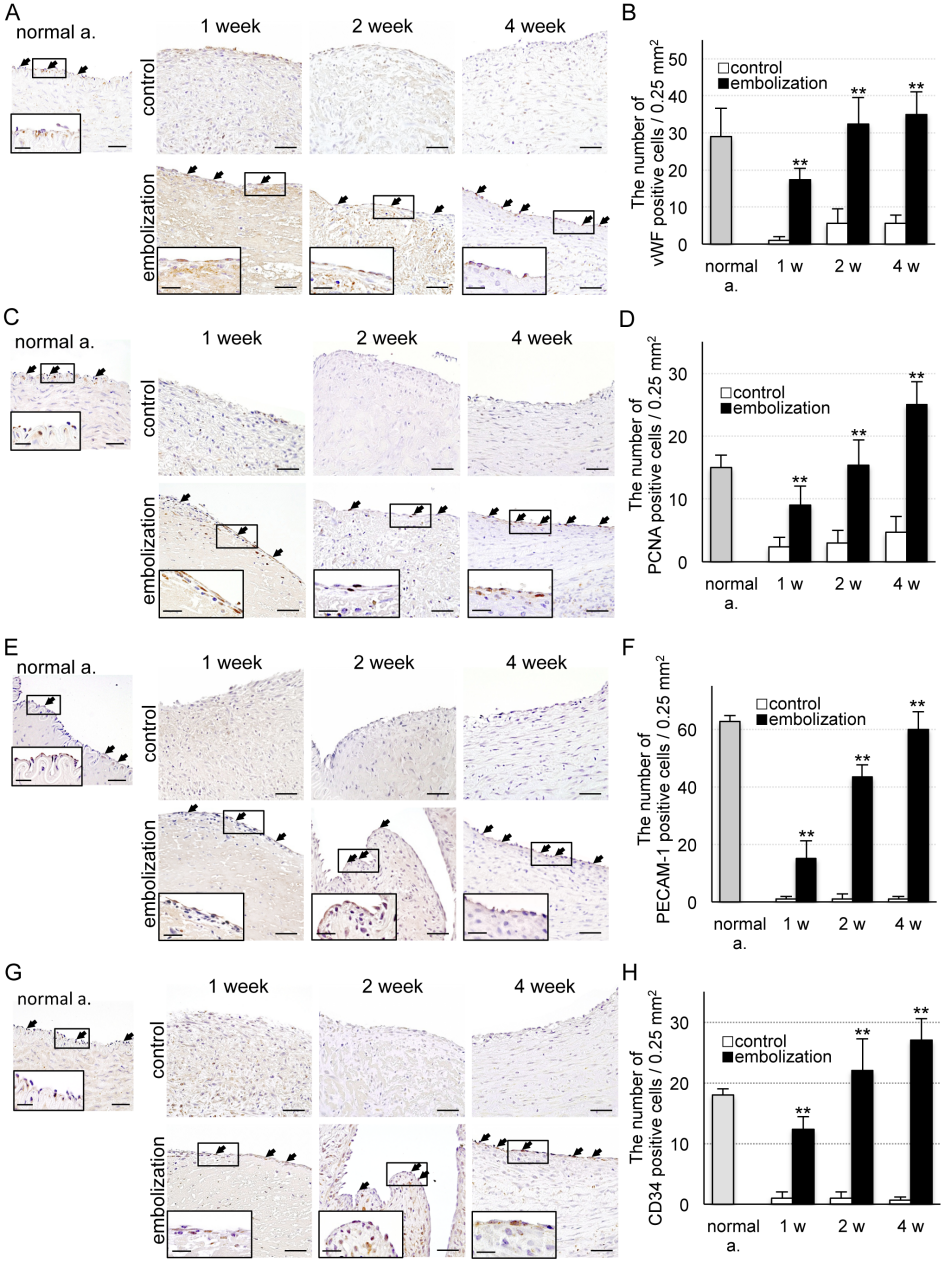
Immunohistochemical analysis of proliferating cells. (**A**) Photomicrographs of vWF-positive cells 1, 2 and 4 weeks after surgery in the embolization and control groups. Scale bars = 50, 20 µm (enclosure). (**B**) Number of vWF-positive cells. (**C**) Photomicrographs of PCNA-positive cells 1, 2 and 4 weeks after surgery in the embolization and control groups. Scale bars = 50, 20 µm (enclosure). (**D**) Number of PCNA-positive cells. (**E**) Photomicrographs of PECAM-1-positive cells 1, 2 and 4 weeks after surgery in the embolization and control groups. Scale bars = 50, 20 µm (enclosure). (**F**) Number of PECAM-1-positive cells. Bars = 50, 20 µm (enclosure). (**G**) Photomicrographs of CD34-positive cells 1, 2 and 4 weeks after surgery in the embolization and control groups. Bars = 50, 20 µm (enclosure). (**H**) Number of CD34-positive cells. Data are mean ± SEM of three swine in each group. *P<0.05, **P<0.001, compared with the control group.

We detected the normal artery (red dotted line) and vein (blue dotted line), thrombosis (yellow dotted line), proliferating tissue (black dotted line) and evaluation area (green enclosure) using low magnification images of AZAN-stained sections (Figure 3A). In H&E stained sections, lining cells were observed in the neck of the aneurysm in the coil embolization group, but weak and easily detachable tissue was detected in the control group (Figure 3B). One week after coil embolization, cells lined only the margin of the proliferating tissue, but at 2 and 4 weeks covered the proliferating tissue. In the coil embolization group, AZAN staining showed proliferation of collagen fibers under the lining cells (Figure 3C). Next, we examined the lining cells using immunohistochemistry. Two weeks after coil embolization, the number of von Willebrand factor-stained cells (i.e. endothelial cells) had almost returned to normal (*P*<0.001, Figure 4A, B). In the coil embolization group, the number of PCNA-stained cells (i.e. proliferating cells) was significantly greater than the control group (*P*<0.001, Figure 4C, D). The number of PECAM-1-stained cells (i.e. those expressing cell adhesion molecules) increased gradually but steadily over time in the coil embolization group (*P*<0.001, Figure 4E, F). The number of CD34-stained cells (i.e. proliferating endothelial cells) increased significantly over time in the coil embolization group (*P*<0.001, Figure 4G, H).

### Morphological Findings

**Figure 5:**
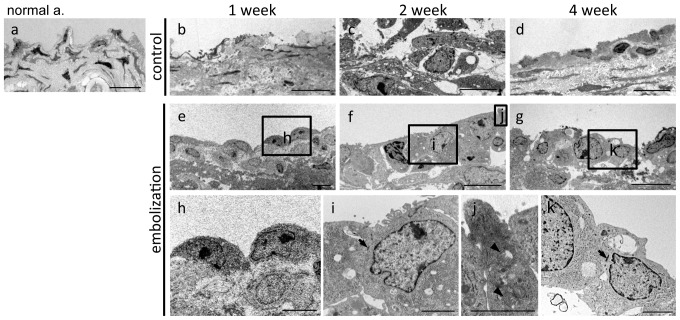
Ultrastructural examination of proliferating cells. Transmission electron microscopy of: a) normal artery; e–k) in the embolization and b–d) control groups 1, 2 and 4 weeks after surgery. Arrow: tight junction; arrowhead: mitochondrion. Scale bars = 10 µm (a–g), 2 µm (h–k).

Finally, TEM was performed to study the cellular morphology of the proliferating tissue. In the control group, no lining cells were observed covering the former aneurysmal orifice at any time (Figure 5b–d). In contrast, lining cells with immature cell structures were detected over the orifice in the coil embolization group (Figure 5e–k). Only the cytoplasm, nucleus, and nucleolus were observed 1 week after coil embolization (Figure 5e, h), while organelles (such as mitochondria, rough endoplasmic reticulum, and ribosomes) and cell junctions were detected 2 and 4 weeks after coil embolization (Figure 5f, g, i–k).

## Discussion

Recanalization of an embolized aneurysm is an independent longitudinal prognostic factor. Several studies have shown that stable aneurysmal thrombosis is associated with the formation of an endothelium-lined layer of connective tissue between the aneurysm and parent artery months after embolization [8], [9], [16], [17]. However, these findings do not show when and how early the layer of new endothelium develops over the neck of the obliterated aneurysms, and no time-dependent comparison of the histologic changes has been described. In this study we observed the proliferation of endothelial cells in a sidewall-type aneurysm model in the first 4 weeks after embolization.

There are many histopathological reports of small animal aneurysm models, such as mouse, rat and rabbit [10], however, in these animals, the vessel structure and coverage of endothelial cells are different to those seen in humans. The small animal models can only be used to study small aneurysms and it is difficult to use devices such as microcatheters and guidewires designed for humans. Moreover, the type of microglia, the inflammatory response, and the structure of blood vessels differ fundamentally from humans as a consequence of differences between the human and rodent immune systems [18]–[21]. Surgical construction of experimental aneurysms in large animals was first described in the dog in the mid 1950s and, more recently, in swine [22]. Their large blood vessels make surgical construction, pathophysiological investigation, and subsequent endovascular or surgical treatment of aneurysms considerably easier than in small animals. Swine have been proposed as a particularly useful model because of similarities in the swine and human coagulation systems [14], [23], [24]. In addition, large animal models have been extensively used for preclinical testing of endovascular devices [9], [14], [23]–[30], and similarly good outcomes can be achieved in large animals and humans – reflected in the findings of this study.

Previous studies have described endothelialization using macroscopically, using H&E, elastic and trichrome staining [9], Masson trichrome and reticulin staining [16], scanning electron microscopy and TEM [9], [17]. At the neck of the aneurysm, the fresh thrombus was progressively replaced by fibrous tissue, growing inwards from the margins. Fibrous obliteration of the neck occurred more rapidly than resolution of the intraluminal blood clot and was present, together with endothelial overgrowth, as early as 14 days after coil embolization [16]. Our study showed a similar pattern of endothelial cell growth.

We detected vWF- and PCNA-positive cells in the proliferating tissue, suggesting that they were proliferating endothelial cells. Moreover, the time-dependent increase in the number of PECAM-1 positive cells, the presence of only cytoplasm, a nucleus, and a nucleolus at 1 week after coil embolization, and the observation of mitochondria, rough endoplasmic reticulum, ribosomes, and tight junctions at 2 and 4 weeks after coil embolization, suggest that the proliferating cells are maturing.

Here, we present our findings from the sidewall-type aneurysm model, but we have also developed a terminal-type aneurysm model, which displays long-term patency [13]. Because the hemodynamic characteristics of sidewall- and terminal-type aneurysms differ, we also plan to study endothelial cell proliferation in the terminal type aneurysm model. Moreover, future studies could also examine the influence of bioactive and hydrophilic coils, balloons, stents, and antiplatelet drugs on embolism and the proliferation of an endothelial lining in both models. We detected an endothelium-lined layer of connective tissue between the aneurysm and parent artery after embolization. In future, the use of bioactive and hydrophilic coils may further promote endothelial cell proliferation, allowing further improvements in the coil embolization of cerebral aneurysms.

## Conclusion

This study demonstrates proliferation of immature endothelial cells over the aneurysmal orifice after coil embolization in a sidewall-type aneurysm model. Our findings suggest that endothelial cells create septal tissue between the systemic circulation and the aneurysm, and then proliferate. Moreover, the proliferated endothelial cells mature over time. This is the first study to evaluate the temporal changes in tissue proliferation in a swine experimental aneurysm model.
